# Community‐based models of alcohol and other drug support for First Nations peoples in Australia: A systematic review

**DOI:** 10.1111/dar.13477

**Published:** 2022-05-11

**Authors:** Jacynta Krakouer, Melissa Savaglio, Karinda Taylor, Helen Skouteris

**Affiliations:** ^1^ Health and Social Care Unit, School of Public and Preventive Medicine Monash University Melbourne Australia; ^2^ First Peoples' Health and Wellbeing Thomastown and Frankston Australia; ^3^ Warwick Business School University of Warwick Conventry UK

**Keywords:** Aboriginal and Torres Strait Islander peoples, alcohol and other drug support, community‐based treatment, First Nations peoples, substance abuse treatment

## Abstract

**Issues:**

The transgenerational impacts of colonisation—inclusive of dispossession, intergenerational trauma, racism, social and economic exclusion and marginalisation—places First Nations peoples in Australia at significant risk of alcohol and other drug (AOD) use and its associated harms. However, knowledge and evidence supporting community‐based AOD treatment for First Nations adults is limited. Therefore, this review aimed to examine the impact and acceptability of community‐based models of AOD support for First Nations adults in Australia.

**Approach:**

A systematic search of the empirical literature from the past 20 years was conducted.

**Key Findings:**

Seventeen studies were included. Nine studies evaluated the program's impact on substance use and 10 studies assessed program acceptability (two studies evaluated both). Only three out of nine studies yielded a statistically significant reduction in substance use. Acceptable components included cultural safety, First Nations AOD workers, inclusion of family and kin, outreach and group support. Areas for improvement included greater focus on holistic wrap‐around psychosocial support, increased local community participation and engagement, funding and breaking down silos.

**Implications:**

Culturally safe, holistic and integrated AOD outreach support led by First Nations peoples and organisations that involves local community members may support First Nations peoples experiencing AOD concerns. These findings may inform the (re)design and (re)development of community‐based AOD services for First Nations peoples.

**Conclusion:**

There is a limited evidence‐base for community‐based AOD programs for First Nations peoples. First Nations‐led research that is controlled by and co‐produced with First Nations peoples is necessary to extend our understanding of community‐based programs within First Nations communities.

## Introduction

Colonisation, as an ongoing structure, not an event, has created conditions that have impacted the health and wellbeing of First Nations peoples in settler‐colonies where the coloniser has come to stay, rather than to extract and leave [1]. In Australia, First Nations peoples continue to assert their sovereignty [2] and articulate the need to address the harms caused by colonisation, racism, discrimination, and economic and social marginalisation [3]. Dismantling systems of oppression and improving the health and wellbeing of First Nations peoples' and communities requires concerted effort.

First Nations peoples in Australia experience significantly poorer health outcomes compared to non‐Indigenous Australians [4]. Harmful alcohol, tobacco and other drug use (herein referred to as AOD or substance use) among First Nations peoples is a key concern contributing heavily to this health disparity. AOD use among First Nations peoples is tied to an explicitly racist history. For example, Western alcoholic spirits (such as gin) and wine were introduced by White settlers to First Nations peoples in Australia, and subsequently used as a mechanism by White settlers to control and coerce the “native population” [5]. Furthermore, when forced dispossession resulted in limited access to traditional food sources, settlers enticed First Nations peoples into townships and colonies with alcohol and tobacco; First Nations labour was paid with alcohol and tobacco; and alcohol was used by White settlers “in barter for sexual favours from Aboriginal women” [5]. Colonial constructions of the “drunken Aborigine” have also stemmed from this problematic history and persist in contemporary stereotypes about First Nations peoples in Australia [5, 6]. This colonial history—of dispossession, trauma and violence—continues to influence substance use within First Nations communities today.

First Nations peoples have asserted the importance of healing past traumas by ensuring culturally appropriate responses to AOD treatment in Australia. In Australia, AOD treatment can take varying forms, from harm minimisation, community‐based brief intervention within primary health‐care settings, such as general practitioner (GP) clinics, or treatment within specialist AOD settings, such as residential rehabilitation or withdrawal services [7]. These varying forms of AOD treatment are needed in a context where the reported rate of illicit drug use for First Nations peoples in Australia (27%) is almost double the rate of non‐Indigenous Australians (15%) [8, 9]. Tobacco use accounts for 23% of the health gap between First Nations peoples and non‐Indigenous Australians, with First Nations women (44%) more than 3.5 times more likely to smoke during pregnancy than non‐Indigenous women (12%) [7, 8, 9]. The prevalence rate of tobacco smoking remains high for First Nations peoples despite declining in other Australian populations. While complete abstinence from alcohol among First Nations peoples in Australia is higher than the non‐Indigenous population [10], other patterns of alcohol use indicate growth—in part due to a colonial history that segregated First Nations peoples from the White Australian population and banned First Nations peoples from entering establishments such as pubs to consume alcohol [11, 12]. First Nations peoples are more likely to engage in risky alcohol consumption, where consumption of alcohol that exceeds lifetime risk has increased from 14.7% in 2014 to 18.4% in 2019 [10]. However, in Australia, there are insufficient dedicated AOD services designed for, and led by, First Nations peoples [13], where a range of socioeconomic, and cultural, barriers to accessing culturally‐safe AOD treatment for First Nations peoples remains concerning [14, 15]. For example, only 17% of all clients seeking AOD treatment services in Australia are First Nations peoples [10]. Furthermore, non‐Indigenous AOD services impose numerous linguistic, cultural and logistical barriers to the access of AOD treatment [15, 16], while AOD services lack funding to adequately support First Nations peoples with complex medical, psychological, sociocultural and physical comorbidities associated with their AOD use [10, 17], which further compounds AOD service provision for First Nations peoples.

In recognising the need to address systemic barriers to treatment access and suitability, AOD treatment for First Nations peoples over the past 20 years has started to focus more on community‐based treatment and support, often delivered within Aboriginal Community‐Controlled Organisations or Aboriginal Community‐Controlled Health Organisations/Services. Community‐based AOD treatment options are varied, encompassing AOD prevention, harm reduction, treatment and aftercare. Treatment most commonly includes brief intervention and motivational interviewing approaches to help people change their AOD use behaviours, typically delivered via outreach (i.e. community mobile treatment and home visits) by an AOD health worker or AOD counsellor [18]. Other forms of community‐based support may include: screening for AOD use; community group support programs; the provision of medication and prescription drugs, such as methadone and champix, to treat opioid and nicotine dependence respectively; the provision of nicotine‐replacement patches or gum to reduce tobacco consumption; supervised injecting rooms for safe injection of substances; and outreach or case‐management support by an AOD worker [18]. Ultimately, community‐based AOD programs attempt to address existing barriers to treatment access and engagement, as support predominantly occurs in one's home or community whereby the community becomes the “treatment facility” [18].

Specifically, alcohol screening and brief intervention (SBI) has been most commonly used by health‐care workers and GPs to detect and treat AOD use among First Nations peoples [19, 20]. Sometimes, training is provided to GPs in alcohol SBI using customised tools to determine treatment factors, such as a patient's alcohol consumption levels, feelings towards alcohol use and its impacts, and readiness for change [20]. However, research has found that health practitioners, including GPs, are reticent to utilise interventions such as SBI for a range of reasons, including concerns about ‘damaging patient rapport’, feeling as though AOD SBI is not within the core remit of a GP's role (particularly when patients are presenting for another health and/or wellbeing condition) and a lack of appropriate referral pathways [19]. It has also been found that Aboriginal health workers are reluctant to conduct alcohol screening with First Nations patients, preferring GPs to conduct screening, yet GPs have identified a range of constraints to the use of alcohol SBI, including a lack of time and “client resistance” to alcohol SBI [20].

However, past research regarding community‐based AOD treatment options, including SBI, has been predominately led by non‐Indigenous researchers. Tools for GP use in SBI are rarely designed by First Nations peoples, but rather, designed by non‐Indigenous research teams, thus raising questions about the cultural‐appropriateness of these tools [20]. Nonetheless, expectations relating to First Nations‐led research have been strengthened over time, particularly within research ethics processes. For example, the Aboriginal Health and Medical Research Council of New South Wales, which is the peak body for Aboriginal Community‐Controlled Health Services in New South Wales [21], aims to ensure that research affecting First Nations peoples and communities is developed in a culturally appropriate way and of good ethical standard. First Nations community control, holistic and comprehensive approaches to health and wellbeing, and First Nations cultures and sovereignty, form part of their core principles [21]. At this juncture, it is important to synthesise the research evidence relating to community‐based AOD programs for First Nations peoples to showcase what may have changed over time, and what the evidence states about community‐based AOD programs for First Nations peoples.

The current knowledge and evidence‐base supporting community‐based AOD programs for First Nations adults is limited. To the authors' knowledge, there has been no synthesis of the impact of community‐based AOD programs specifically for First Nations adults in Australia, with previous reviews focusing on other types of services (i.e. residential rehabilitation) [22], the youth population [23, 24] or combining Australian and international literature [18, 25, 26]. The extent of their acceptability (i.e. how well interventions are received/deemed useful or valuable by its end users) is also unknown. A synthesis is warranted to inform current practice, service delivery and potentially improve AOD‐related outcomes for First Nations adults. Therefore, this review aimed to examine the impact and acceptability of community‐based models of AOD support for First Nations adults in Australia.

## Method

### Design and protocol registration

A systematic review was conducted in line with the Preferred Reporting Items for Systematic Reviews and Meta‐Analyses (PRISMA) statement guidelines. The protocol was registered with PROSPERO.

### Search strategy

The empirical literature was systematically searched for studies that evaluated the impact and/or acceptability of a community‐based AOD program for First Nations peoples in Australia. Six electronic databases were searched: PsycINFO, MEDLINE, CINAHL plus, Australian Indigenous HealthInfoNet, Indigenous Collection and Health Collection, combining keywords related to: First Nations peoples (i.e. ‘Aboriginal’, ‘Indigenous’) and ‘substance use’ (see Appendix). The search strategy incorporated medical subject heading (MESH) search terms and keywords, which were customised to each database as needed. The reference lists and citations for included articles were also examined.

### Inclusion and exclusion criteria

Studies were included in the review if they met the following inclusion criteria: (i) participants were First Nations adults experiencing substance misuse; (ii) the study was conducted in Australia; (iii) the study quantitatively or qualitatively evaluated a community‐based model of AOD treatment (e.g. delivered via outreach, in homes, community centres, community health services) that was specifically designed for and delivered to First Nations adults to address substance use; (iv) study outcomes included impact on substance use and/or acceptability of the intervention; and (v) the study was published in English in a peer‐reviewed journal from January 2000 to August 2021 inclusive. Given the relatively limited empirical literature on AOD program evaluations for First Nations peoples in Australia [27], studies were identified from the past 20 years to encompass the most relevant evidence to inform current practice.

Studies were excluded if: (i) they did not examine the impact of the intervention on the clients themselves (e.g. health workers' outcomes); (ii) they described an intervention or model of support without any evaluation (i.e. protocol, descriptive studies); (iii) they examined the impact and/or acceptability of a mainstream service that was not specifically targeted to First Nations clients; (iv) they evaluated AOD health promotion strategies or policy/government initiatives (i.e. limits to supply, alcohol management plans); and (v) the program was delivered in an acute, inpatient or residential setting, as these settings were not considered ‘community‐based’ and have been previously synthesised [22].

### Study selection

Two researchers independently screened and excluded studies based on titles and abstracts. For articles not excluded, the full‐text versions were sourced and assessed for inclusion by the same two researchers. The interrater agreement (proportion of studies given the same rating by the two researchers) during the title and abstract stage and full‐text stage was 0.92 and 0.95, respectively.

### Data extraction and synthesis

Summary tables were created to extract data from the included studies (see Table 1). Data extracted included: state where the study was conducted; study design; participant characteristics (i.e. size, age, gender, type of substance use); intervention characteristics; outcomes and measures; and findings. As there was significant heterogeneity across studies in terms of study design, outcomes, measures and reported data, meta‐analysis was not possible [28]. Instead, the findings were categorised and narratively described according to type of study (i.e. impact versus acceptability).

**Table 1:** Core intervention components

Component	Explanation
Culturally safe	Support delivered “on Country” Ensure privacy and sensitivity Restoring cultural connections and community networks Inclusion of family and kin where possible in treatment Embed opportunities for engagement with culture within programs, including cultural practice such as yarning
Local community involvement	Delivered by local First Nations community members, leaders, or Elders, or health workers Educate and inform local community members of services Trust and rapport between service providers and clients Increase program uptake throughout the community Ensures cultural safety
Wrap‐around psychosocial support	Holistic and integrated support Addressing factors perpetuating substance use Alleviating the psychosocial stressors/consequences associated with substance use Break down silos that exist between alcohol and other drug and other services (i.e. housing, supported employment, counselling, family violence support, financial aid)
Outreach	Reduce barriers to treatment access Support delivered in the community, home visits etc. Increased engagement
Group work/activities	Distraction or aversion from engaging in substance use Socialisation and support from positive role models Storytelling, yarning, sharing of experiences Practice strategies and problem solving Target existing local community groups (i.e. arts, parenting)

### Quality assessment

The methodological quality assessment of included studies was conducted using the Aboriginal and Torres Strait Islander Quality Appraisal Tool [29]. This is a tool developed by First Nations peoples to appraise the quality of research conducted with First Nations peoples, focusing on First Nations epistemologies, values and principles for ethical research. The tool was developed using the Nominal Group and Delphi techniques by a group of senior Aboriginal and Torres Strait Islander researchers [for more information see Harfield *et al*. 29]. It comprises 14 questions to assess the rigour of the study design and appropriateness of methods, rated as either ‘yes’, ‘partially’, ‘no’ or ‘unclear’. Studies that scored ‘yes’ for at least 70% of their assessment criteria were categorised as high quality as they aligned with the values and principles for ethical research from a First Nations lens. Studies that fulfilled 50% to 70% of the criteria were considered medium quality, and studies that addressed less than 50% of the criteria were classified as low quality. Consensus was achieved through a cooperative discussion between two researchers, where the interrater agreement was 0.94.

## Results

### Search yield

**Figure 1:**
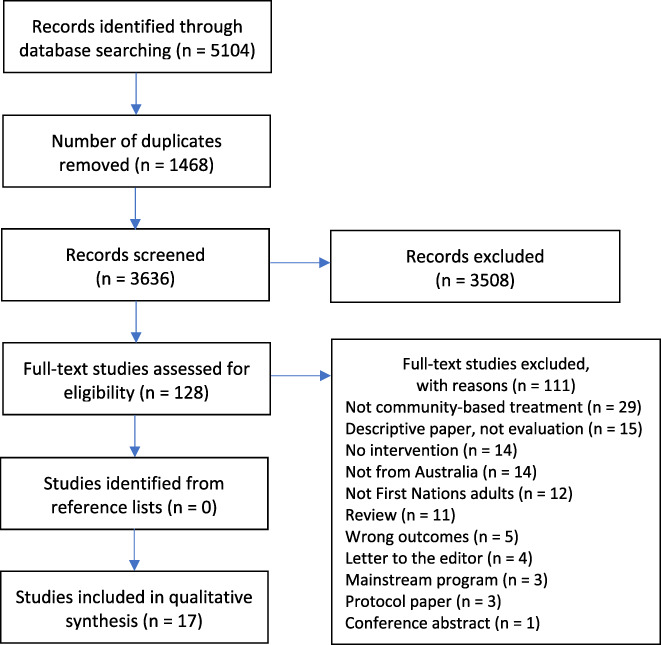
PRISMA Flowchart of Study Selection and Screening

The stages of study selection are summarised in the PRISMA flowchart presented in Figure 1. The search of the six electronic databases identified a total of 5104 studies. There were 3636 studies screened for eligibility at the title and abstract stage, followed by 128 full‐text studies. A total of 17 studies were deemed eligible and included in this review.

### Quality assessment

The quality assessment of the 17 studies is presented in Table S1 (Supporting Information). The majority of studies (*n* = 11, 64%) were classified as medium quality [19, 20, 27, 30, 31, 32, 33, 34, 35, 36, 37], three studies (18%) were high quality [38, 39, 40] and three studies (24%) were low quality [41, 42, 43] due to a lack of reporting or alignment with the criteria [29]. Higher quality research was developed, led and conducted by First Nations peoples (i.e. First Nations researchers, leadership, governance, and inclusive community consultation and engagement to guide research activities). In studies consistently adopting strengths‐based approaches, the research was of clear benefit to First Nations communities, and often demonstrated local workforce capacity building. Collaborative partnerships between Aboriginal Community‐Controlled Organisations or Aboriginal Community‐Controlled Health Organisations and universities were pivotal to support this research. However, First Nations peoples need greater control over the collection and management of research materials/data and transparency in reporting of research methods (i.e. development of Collaborative Research Agreements, ensuring First Nations peoples have ownership of intellectual and cultural property).

### Summary of studies

A summary of the 17 studies is presented in Table S2 (Supporting Information). Nine studies evaluated program impact [32, 33, 34, 35, 36, 37, 40, 42, 43] and 10 studies assessed program acceptability [19, 20, 27, 30, 31, 32, 34, 38, 39, 41]. There were seven qualitative studies [19, 20, 27, 30, 31, 38, 41], two studies that adopted mixed methods [32, 39], one pre‐post study [33], one cross‐sectional study [34] and six controlled studies: three quasi experimental without randomisation to groups [35, 42, 43] and three randomised controlled studies [36, 37, 40]. Studies were predominantly conducted in New South Wales (*n* = 7) [19, 27, 30, 33, 34, 39, 41], followed by the Northern Territory (*n* = 4) [35, 38, 40, 43].

### Participant characteristics

Participants identified as either Aboriginal, Torres Strait Islander, Indigenous or First Nations peoples. These terms were used interchangeably to refer to First Nations peoples whose lands are situated in Australia. The heterogeneity of Nation groups to which participants belonged was rarely reported; one study specified that participants were Yolngu peoples from north‐eastern Arnhem Land [38]. The mean sample size was 87 (SD = 73), ranging from 8 to 263, excluding one outlier sample of 702 participants [42]. The proportion of participants who identified as male was 36%. The low representation of males may be attributed to the four studies that focused exclusively on women [27, 34, 36, 41], whereas no studies included samples of 100% men. Only seven studies reported the average age of participants [27, 33, 34, 35, 37, 40, 42], of which the overall mean age was 34.56 years (SD = 5.23). The remaining 10 studies did not report the sample's mean age [19, 20, 30, 31, 32, 36, 38, 39, 41, 43]. Studies evaluated interventions targeting alcohol dependence (*n* = 7) [19, 20, 30, 31, 35, 38, 39], tobacco (*n* = 5) [34, 36, 37, 42, 43], varied substances (*n* = 3) [27, 33, 41], opioids (*n* = 1) [32] or comorbid alcohol and cannabis use (*n* = 1) [40].

### Characteristics of interventions

Each intervention is described in Table S2. On average, interventions were delivered weekly for 6 months (*M* = 25.63 weeks, *SD* = 21.97). However, there was significant variation in the duration and intensity of support, ranging from one session of brief intervention [20, 43] to over 12 months of engagement, with daily contact for the more intensive outreach programs [30, 38]. Five interventions included a group component [27, 31, 33, 39, 41]. Interventions were predominantly delivered by First Nations health workers (*n* = 11) [19, 20, 33, 34, 36, 37, 38, 39, 40, 42, 43], GPs (*n* = 6) [19, 20, 27, 32, 33, 39], AOD counsellors (*n* = 5) [27, 30, 31, 32, 41] and/or community Elders (*n* = 1) [31].

### Intervention descriptions

All interventions were community‐based programs delivered via outreach (i.e. home‐visits, community meetings, existing groups) or at local community centres or community health services. Four of the 17 studies evaluated a group program that focused on members discussing their experiences, psychoeducation, storytelling, interactive discussions and skills training to address their substance use. The Hero to Healing Drink Driving program (The Hero to Healing Drink Driving program was delivered by community Elders and local AOD workers) supported members to change external or other lifestyle factors to reduce their alcohol abuse [31]. The women's support group for substance use comprised of informal conversations, arts and crafts, recreational and educational activities [27]. The Softy Entry Approach [41] and Alcohol Awareness program [39] had AOD counsellors or health workers attend existing First Nations community events, gatherings or support groups (i.e. art, cooking, storytelling, Alcoholics Anonymous, etc.) to engage people in discussions around substance use. Four studies assessed Brief Intervention, which involved screening, brief advice, referral to specialist support, counselling or brief motivational interviewing delivered by First Nations health workers and GPs [19, 20, 40, 43].

Two studies evaluated smoking cessation support for pregnant women during antenatal outreach visits, which included screening and psychoeducation [34, 36]. Two studies delivered intensive multi‐component smoking cessation programs, comprising motivational interviewing, support groups and community‐based events [42] or therapeutic support, pharmacotherapy and case management [37]. Five programs incorporated practical wrap‐around support to address key issues perpetuating substance use, respond to individuals' needs as they arose, and facilitate rehabilitation alongside pharmacological treatment [27, 30, 32, 35, 38]. This included support with housing, family violence, child protection, finances, employment, vocational training, restoring family, social and cultural connections, mental health, physical health or transportation to and from appointments. The final study assessed the Aboriginal‐adapted Community Reinforcement Approach where both Aboriginal and non‐Indigenous Health Care Providers delivered therapeutic individual and group outreach support to facilitate psychoeducation and skill acquisition to address alcohol‐related problems [33].

### Intervention impact

Nine studies evaluated the effect of the program on participants' substance use via self‐report measures [32, 33, 34, 35, 36, 37, 40, 42, 43]. Only three studies found a statistically significant reduction in substance use: two focused on tobacco and one examined varied substances. Specifically, Campbell *et al*. [42] observed a significant reduction in smoking prevalence and in the number of cigarettes smoked weekly among intervention participants compared to controls. Following a brief intervention for smoking cessation, 15% of intervention participants reported quitting and 76% reported reduced tobacco consumption in comparison to control participants (1% and 51%, respectively) [43]. The Aboriginal‐adapted Community Reinforcement Approach to treatment yielded significant reductions in alcohol, cannabis and amphetamine use, yet not in tobacco, cocaine, inhalant, injecting, sedative or opiate use following participation [33].

In contrast, the remaining three smoking cessation programs did not yield any significant reductions in smoking in comparison to usual care [34, 36, 37]. Similarly, there were no significant reductions in cannabis or alcohol use following brief intervention and motivational care planning [40] or the Grog Mob program [35]. Finally, only 10% of participants of The Way Out program had successfully ceased opioid use [32]. The authors proposed that small sample sizes and lack of engagement may have led to reduced power to identify significant improvements.

### Intervention acceptability

Nine of the 17 studies evaluated intervention acceptability via qualitative methods from the perspectives of clients (i.e. those receiving the intervention) and/or health workers (i.e. those delivering the intervention) [19, 20, 27, 30, 31, 32, 34, 38, 39, 41]. Table 1 presents a summary of the core intervention components.

#### Acceptable components

Seven of the 10 programs were generally well accepted by participants [27, 30, 31, 32, 34, 38, 41]. Support that was perceived as culturally safe was consistently most acceptable to First Nations peoples. Programs delivered by First Nations health workers facilitated the development of trust and rapport with clients, particularly if they were part of the same local First Nations community [27, 30, 31, 32, 38]. A key enabler to program uptake was having a large local First Nations community presence, by engaging, educating and developing relationships with key community members, leaders and Elders [31, 32]. Clients described their health workers and AOD counsellors as non‐judgemental, approachable, genuine, friendly and easy to talk to, which helped to build rapport [20, 32, 38]. Community outreach programs for alcohol dependence were valued by clients for improving treatment access and for their focus on restoring links and connections with family, kin, cultural and community networks [30, 38]. Finally, participants of the Hero to Healing alcohol support program appreciated that the group was held ‘on Country’ at a location chosen by participants away from the community to ensure the safety and privacy of sensitive content [31].

Group components were valued by clients as they provided: an opportunity to share experiences; relatable and practical strategies; a sense of stability; a distraction from engaging in substance use; relaxation, socialisation and support from positive role models; and were delivered in a culturally safe environment [27, 31, 38]. Clients valued programs that incorporated practical and integrated wrap‐around support to address key issues perpetuating substance use and facilitate rehabilitation following detoxification (e.g. housing, family violence, child protection, financial support, employment, social and cultural connections, mental health) [27, 30, 38].

### Areas for improvement

Participants across all nine studies evaluating acceptability provided suggestions to enable programs to better meet the needs of First Nations communities. Brief intervention for alcohol was generally not well received by clients and health workers [19, 20, 41]. No participants agreed to one‐on‐one intervention following group outreach education sessions [39]. Furthermore, some First Nations health workers expressed reluctancy to question clients about their alcohol consumption and preferred if it was someone outside of their community, noting that it could potentially offend clients, damage rapport, elicit shame and that clients often ‘fudged’ their responses [20]. Similarly, some clients were reluctant to engage in group treatment due to the potential leakage of personal information to the wider First Nations community [30]. GPs expressed concern about the possibility of uncovering the complexity of clients' broader psychosocial problems associated with drinking, which they did not have the time, capacity or expertise to address [19, 20]. Outreach services need to increase participation, guidance and engagement of local First Nations peoples as champions, leaders or staff (i.e. community Elders, peer workers) to promote trust and engagement [27, 30, 31, 32, 38]. More regular facilitation and embedding of culture would increase acceptability. For example, women's support group participants desired more opportunities to share stories, in line with First Nations traditions and practices of storytelling [27].

Practitioners identified a lack of holistic follow‐up support or referral options (i.e. alcohol detoxification services, counselling, practical support, transportation, etc.) as a key barrier; without this wrap‐around support to address broader issues, health workers perceived brief intervention to be of little benefit to clients. Holistic and integrated outreach approaches that address psychosocial factors associated with substance use (i.e. housing, employment, counselling, family violence, financial support) was recommended to facilitate change [30, 31]. Other identified drawbacks included difficulty encouraging some clients to attend the clinic for follow‐up treatment [38], lack of 24‐hour support (as opposed to the residential or inpatient setting) [30], limited funding or strict funding guidelines [41], and that AOD practitioners and services often operated in silos from the broader organisation or other relevant services [31, 38].

## Discussion

There is little knowledge regarding the effectiveness and acceptability of community‐based models of AOD treatment for First Nations adults in Australia. This review aimed to examine the impact and acceptability of community‐based models of AOD support for First Nations adults in Australia. Seventeen studies were identified; nine studies evaluated their impact on clients' substance use and nine studies evaluated intervention acceptability.

The current findings indicate that there is a limited evidence‐base for community‐based AOD programs for First Nations peoples. Previous local literature has also noted the dearth of evaluations of AOD interventions that are tailored for First Nations populations [44], resulting in a lack of culturally appropriate care [44]. Only three of the nine studies that evaluated the program's impact on clients' substance use found a statistically significant reduction in substance use following the intervention (via uncontrolled or controlled evaluation). This comprised a multi‐component tobacco control program [42], brief intervention for smoking cessation [43] and the Aboriginal‐adapted Community Reinforcement Approach in which Aboriginal and non‐Indigenous health‐care providers delivered therapeutic individual and group outreach support [33]. The lack of effectiveness of the remaining programs, particularly those involving brief intervention and motivational interviewing, is concerning given that such programs are the most common type of community‐based AOD treatment. Small sample sizes and lack of engagement were proposed by authors as potential attributions for non‐significant findings [32, 40]. First Nations peoples' mistrust of research has been previously highlighted [44], resulting in a reliance on purely descriptive studies [22] and a limited evidence base to support the effectiveness of community‐based AOD treatment for First Nations peoples in Australia [45]. Therefore, higher quality evaluation of such programs is warranted to enhance the evidence‐base, alongside greater focus on adapting and tailoring community‐based models of AOD support that demonstrate improved efficacy for First Nations peoples.

Given the lack of effectiveness studies, including acceptability studies was necessary to identify factors that may facilitate engagement, retention and positive outcomes among First Nations adults, from the perspective of the end‐users themselves. The majority of programs were considered acceptable models of community‐based AOD programs for First Nations peoples. The most common component was ‘culturally safe, appropriate, or responsive’, which included a focus on cultural engagement, restoring cultural connections, First Nations‐specific resources, support delivered ‘on Country’, aligning with First Nations peoples' cultures, values and traditions, and involving the local community. These cultural elements have been shown to positively influence the patient–practitioner relationship, which is a key predictor of treatment effectiveness [46]. Improving access to services through the provision of culturally acceptable services and models of care is crucial to improving AOD‐related outcomes among First Nations adults [47]. Indeed, the emphasis on culture is key to ensuring the success of such programs. Specifically, First Nations clients valued when programs were delivered by local First Nations community members, leaders or Elders [30, 31]. Internationally, models of support that involve the local community facilitate trust and rapport, encouraging engagement, program uptake and promoting awareness of the service across diverse, international First Nations communities [48]. While it is acknowledged that in practice many First Nations peoples in Australia will receive treatment from non‐Indigenous health workers, the current findings highlight that Aboriginal and/or Torres Strait Islander clients perceive that having a worker who is also Aboriginal and/or Torres Strait Islander is generally more acceptable. Practical implications of this may include ensuring that First Nations clients can choose their worker to potentially enhance engagement, and this warrants a greater focus on building the workforce capacity of local First Nations AOD workers and counsellors to increase reach and access to community‐based AOD treatment for First Nations peoples across Australia.

Given that strengths‐based and protective factors for reducing AOD use among First Nations peoples in Australia tends to include cultural, familial, kinship and community members, this kind of holistic approach is the most promising to work towards [49]. Aboriginal Community‐Controlled Organisations provide an opportunity to achieve this: they were built by, and for, First Nations peoples and are intended to embed culturally appropriate service provision, practices and values into their core remit. Therefore, AOD treatment for First Nations peoples in Australia requires a holistic, whole‐of‐community approach, that seeks to include family, kin and cultural obligations or connections and engage community members [50]. This aligns with the perspective of health and wellbeing espoused within the National Aboriginal Health Strategy in 1989 where holistic health and wellbeing encompasses both individuals and communities for First Nations peoples [51]. Self‐determination, inclusive of power and control to deliver culturally appropriate AOD treatment programs, for First Nations communities and organisations is critical.

Finally, limited programs delivered practical and integrated wrap‐around AOD support via outreach to address clients' broader psychological, physical, social and cultural needs [27, 30, 38]. Such factors often contribute to the onset and perpetuation of substance use among First Nations peoples in Australia [10]. It is necessary to ensure that programs can address the impact of the broader societal environment (i.e. racism, oppression, marginalisation and discrimination) which impacts substance use. This approach could also potentially assist in breaking down the silos that exist between AOD services/workers and other services/supports. Therefore, a holistic, wrap‐around, integrated and broader societal approach to AOD support may be feasible, acceptable and effective for First Nations peoples.

### Limitations

The lack of effectiveness studies evaluating community‐based AOD programs for First Nations peoples is a predominant gap in the Australian literature. The literature that is available provides limited evidence to suggest that such models of AOD support can yield statistical and clinically meaningful reductions in substance use among First Nations peoples. However, non‐statistical findings are likely due to small sample sizes and low methodological quality of the effectiveness research. Indeed, engagement of First Nations peoples in research is a longstanding issue, where fear and mistrust of research due to unethical research practices [52] have contributed to high rates of attrition [45]. While it is noted that research being led by First Nations peoples has been enhanced and strengthened in recent years via research ethics processes, greater First Nations community‐control of research (i.e. First Nations‐led research, co‐design, participatory action research and participatory approaches), in line with the principles of ethical research with First Nations peoples in Australia [53], and more transparent reporting of this in study methods, is needed to ensure ethical research and cultural appropriateness [26]. Another limitation related to gender; men were under‐represented in the reviewed studies, with four studies focusing solely on supporting First Nations women. Given that men typically comprise the majority of the AOD treatment population, it is recommended that future research focuses on the implementation and evaluation of community‐based AOD support for First Nations men.

## Implications and Conclusion

These findings provide novel insights into the impact and acceptability of community‐based AOD support for First Nations peoples in Australia. Culturally safe, holistic and integrated AOD outreach support led by First Nations peoples and organisations that involves local community members may yield better outcomes among First Nations peoples experiencing AOD concerns. The findings may be used to inform the (re)design and (re)development of future culturally safe, appropriate and responsive models of community AOD support that incorporate the identified core components. First Nations‐led research, that is controlled by and co‐produced with First Nations peoples, is necessary to ensure reciprocity, respect and culturally appropriate involvement of local community members in the design, development, implementation and evaluation of such programs. These findings may stimulate further research that extends our understanding of the effects of community based health programs within First Nations communities. Best‐practice, culturally‐safe models of AOD outreach care for First Nations peoples are critical, for clients of AOD treatment services, their families and communities.

## Conflict of Interest

The authors have no conflicts of interest.

## Author Contributions

JK drafted the manuscript. MS conducted the systematic search and assisted with drafting the manuscript. KT and HS contributed to the conception, design and aims of the manuscript and provided critical feedback on each draft. All authors approve this version of the manuscript to be published. Each author certifies that their contribution to this work meets the standards of the International Committee of Medical Journal Editors.

## Supporting information


**Table S1**. Quality assessment.
**Table S2**. Summary of studies.

## Appendix A

Search terms

Aboriginal* OR “Torres Strait Islander*” OR Indigenous OR “First People*” OR “First Nation*”

AND

Substance use* OR substance abuse* OR substance misuse OR substance dependenc* OR drug* OR alcohol* OR AOD OR addict*
