# Supplementary material for: Community‐based models of alcohol and other drug support for First Nations peoples in Australia: A systematic review
Source: Drug Alcohol Rev. 2022 May 11;41(6):1418–27. doi: 10.1111/dar.13477 (PMC9542511; doi:10.1111/dar.13477)
Supplement: Supplementary file 1 — Table S1. Quality assessment. Table S2. Summary of studies. [file DAR-41-1418-s001.docx]

**Supporting Information**

**Table S1. Quality assessment**

| **Criteria** | **First author (year)** | | | | | | | | | | | | | | | | |
| --- | --- | --- | --- | --- | --- | --- | --- | --- | --- | --- | --- | --- | --- | --- | --- | --- | --- |
|  | Allan (2011) | Brady (2002) | Brett (2017) | Calabria (2020) | Campbell (2014) | Clifford (2012) | Conigrave (2012) | D’Abbs (2013) | Daly (2021) | Eades (2012) | Fitts (2016) | Ivers (2003) | Lee (2013) | Marley (2014) | Nagel (2009) | Wearne (2005) | Williams (2006) |
| 1. Did the research respond to a need or priority determined by the community? | Y | Y | Y | Y | Y | Y | Y | Y | Y | Y | Y | Y | Y | Y | Y | Y | Y |
| 2. Was community consultation and engagement appropriately inclusive? | P | P | Y | Y | Y | U | Y | U | P | P | P | U | P | U | Y | P | Y |
| 3. Did the research have Aboriginal and Torres Strait Islander research leadership? | N | Y | Y | Y | U | N | Y | Y | U | Y | N | Y | N | Y | Y | Y | Y |
| 4. Did the research have Aboriginal and Torres Strait Islander governance? | N | P | U | Y | N | Y | U | Y | Y | U | Y | U | Y | Y | Y | Y | U |
| 5. Were local community protocols respected and followed? | P | Y | U | U | U | U | Y | U | P | U | Y | U | Y | U | Y | Y | U |
| 6. Did the researchers negotiate agreements in regards to rights of access to Aboriginal and Torres Strait Islander peoples’ existing intellectual and cultural property? | N | N | N | N | N | N | N | N | N | N | N | N | N | N | N | N | N |
| 7. Did the researchers negotiate agreements to protect Aboriginal and Torres Strait Islander peoples' ownership of intellectual and cultural property created through the research? | N | N | N | N | N | N | N | N | N | N | N | N | N | N | N | N | N |
| 8. Did Aboriginal and Torres Strait Islander peoples and communities have control over the collection and management of research materials? | N | N | P | P | P | N | Y | N | P | N | N | N | N | P | P | Y | U |
| 9. Was the research guided by an Indigenous research paradigm? | N | N | P | U | U | N | Y | U | Y | N | U | N | U | U | Y | Y | U |
| 10. Does the research take a strengths-based approach, acknowledging and moving beyond practices that have harmed Aboriginal and Torres Strait peoples in the past? | Y | Y | Y | Y | Y | Y | Y | Y | Y | Y | Y | Y | Y | Y | Y | Y | Y |
| 11. Did the researchers plan to and translate the findings into sustainable changes in policy and/or practice? | U | Y | U | U | U | Y | U | U | U | U | U | U | P | U | U | Y | N |
| 12. Did the research benefit the participants and Aboriginal and Torres Strait Islander communities? | Y | Y | Y | Y | Y | Y | Y | Y | Y | Y | Y | Y | Y | Y | Y | Y | Y |
| 13. Did the research demonstrate capacity strengthening for Aboriginal and Torres Strait Islander individuals? | N | Y | Y | P | P | Y | Y | Y | Y | Y | Y | N | Y | Y | Y | Y | Y |
| 14. Did everyone involved in the research have opportunities to learn from each other? | Y | Y | Y | Y | Y | Y | Y | Y | Y | Y | Y | Y | Y | Y | Y | Y | Y |

N, no; P, partially; U, unclear/not reported; Y, yes.

***Supplementary Table 2.* Summary of Studies**

| **First author (year), state** | **Study design** | **Participant characteristics** | **Intervention description** | **Outcomes and measures** | **Findings** |
| --- | --- | --- | --- | --- | --- |
| Allan (2011), New South Wales | Qualitative  (Acceptability) | Sample size: 149  Mean age: NR  Gender (male): 0%  Substance use: Various, not specified | The Soft Entry Approach:  Delivered in response to high numbers of people referred to community services that did not attend appointments, and that usual entry points into drug and alcohol care posed barriers. Drug and alcohol counsellors aimed to engage people by being present at Aboriginal community events, groups (i.e. Alcoholics Anonymous, Smart Recovery) and gatherings and talking about harmful substance use with community members whenever possible. Counsellors were invited to participate by the Aboriginal organization holding an event to ensure they were vouched for by community leaders. Counsellors focused on creating opportunities to develop a non-stigmatising trustful rapport to facilitate discussion of harmful substance use, and engage people in motivational interviewing.  Duration: 18 month period  Frequency: NR  Delivered by: drug and alcohol counsellors  Mode: outreach, individual or group sessions | Women’s and workers’ experiences of the soft entry approach  Feasibility and acceptability of the program | - participants spoke positively about the soft entry approach and its ability to provide access to drug and alcohol counselling in a non-traditional setting. - women described the counsellor as approachable, sincere, friendly and easy to talk to. - no concerns about the counsellor attending the events - the environment of the groups were a contributing factor in facilitating conversation with the drug and alcohol counsellor - helped to establish trust and rapport with community members - community members told others about the counsellor’s role and that she could be trusted - reducing barriers to treatment programs - described as “outside the treatment tradition” and funding guidelines. |
| Brady (2002), Australian Capital Territory | Qualitative  (Acceptability) | Sample size: n=25  Mean age: NR  Gender (male): NR  Substance use: Alcohol | Brief intervention for alcohol misuse:  Brief intervention comprised a range of strategies including screening, brief advice, referral to specialist support, counselling and brief motivational interviewing. Health workers were trained to screen patients for eligibility for the intervention the AUDIT or two questions about alcohol consumption. Brief intervention was delivered by the GP to those identified through screening as drinking at hazardous or harmful levels. The GPs provided informational resources and delivered motivational interviewing, which included reviewing consumption levels, probable health consequences, the ‘good’ and the ‘not so good’ things about drinking, readiness to change and, if the client showed interest, further advice about ways of cutting down or trying to abstain.  Duration: 12 months  Frequency: one to two sessions  Delivered by: Aboriginal health workers and GPs  Mode: individual, primary health care clinic | Health workers and GPs’ perspective of the acceptability and feasibility of the program | Screening component:   - the health workers found the AUDIT long and intrusive - poor question comprehension, questions needed clarification and were often reworded - clients sometimes ‘fudged’ their responses - increasing comfort with these questions over time   Motivational interviewing:   - seemed appropriate to GPs - non-authoritarian and non-judgemental   Barriers:   - Aboriginal health workers expressed some concerns about questioning clients about alcohol consumptions – they preferred someone outside of the community - small sample size: client reluctance to engage and health worker reluctance - GPs had lack of time, ‘drop-in’ model led to long waiting times - severity of illness and the complexity of the physical, social and psychological problems with which patients present |
| Brett (2017), New South Wales | Qualitative  (Acceptability) | Sample size: 8  Mean age: NR  Gender (male): NR  Substance use: Alcohol | Outpatient detox and support within a community service:  Withdrawal management was always initiated on a Monday or Tuesday to reduce the risk of complications occurring over the weekend. Clients were offered transport to the clinic each morning and the option of a home visit or phone call in the afternoon. The standard detox program lasted for 5 days and involved a daily dispensed, weaning regime of diazepam, along with thiamine, supportive counselling and relapse-prevention planning. The following week, clients received medical follow-up, and counselling or other follow-up as appropriate. They received follow-up phone calls from clinic staff at 2 and 6 weeks. Other health and social needs were also addressed via outreach, home visits and case management (e.g. mental health, physical health, housing, financial support).  Duration: six weeks  Frequency: daily  Delivered by: Aboriginal drug and alcohol counsellors  Mode: outpatient community clinic | Staff and clients’ perspectives of the program | Enablers/acceptable:   - high level of satisfaction - cultural safety and trust - providing outpatient detox through an Aboriginal controlled community health service was preferred over a mainstream service - appreciated that family-focused goals could be achieved - reconnecting with community - individuals within the community to serve as champions to promote new services, e.g. peer support - accessible, streamlined, holistic and integrated support   Barriers:   - shame, group treatment environments could lead to leakage of personal information to the wider community - other drinkers in the home/family environment - home setting meant potential isolation and lack of 24-hour support |
| Calabria (2020), New South Wales | Pre-post  (Impact) | Sample size: n=22  Mean age: 32.54 (12.24)  Gender (male): 58%  Substance use: Various, not specified | Aboriginal-adapted Community Reinforcement Approach:  Drug and alcohol community-based treatment embedded in usual care. Clients received the intervention via outreach. Aboriginal and non-Indigenous Health Care Providers therapists were local people who are known and trusted by the community; alcohol-related harms were discussed sensitively; preference for a detailed, rather than a brief, intervention was honoured; treatment sessions were focused on talking about alcohol-related problems and skill acquisition to address those problems; and follow-up support was provided as necessary.  Duration: six weeks  Frequency: weekly  Delivered by: Aboriginal and non-Indigenous health care workers  Mode: outreach, option for individual or group sessions (87% engaged in group session) | Pre: baseline  Post: 3 months follow-up:  1. Substance misuse: Alcohol, Smoking, and Substance Involvement Test:  a) alcohol  b) tobacco  c) cannabis  d) cocaine  e) amphetamine  f) inhalant  g) sedative  h) opiate  2. Psychological distress: Kessler-5  3. Empowerment: Growth Empowerment Measure  4. Injecting drug use | 1. Significant reductions in alcohol, cannabis, and amphetamine use. No significant reductions in tobacco, cocaine, inhalant, sedative or opiate use. 2. Significant reduction in psychological distress. 3. Significant increase in sense of self-empowerment. 4. Pre: 46%; Post: 40%.   No significant difference. |
| Campbell (2014), Queensland | Quasi-experimental  (Impact) | Sample size: 702  Intervention = 449  Control = 253  Mean age:  Intervention = 37.5  Control = 38.4  Gender (male): 41%  Substance use: Tobacco | Multi-component tobacco control intervention:  A multi-faceted community-based approach to tobacco control, including the following components: (i) event support programs; (i) SmokeCheck brief intervention training to enable health workers to support smokers to quit; (ii) Smoke Rings support program, a 5-week group support program for individuals trying to give up smoking; (iv) workplace smoke-free policy guides; and (v) monitoring of compliance with legislation on tobacco sales.  Duration: NR  Frequency: NR  Delivered by: community-based service providers  Mode: outreach | 1. Smoking prevalence  2. Number of cigarettes smoked weekly | 1. Intervention: 44% to 35%   Control: 40% to 42%  *P* <0.011   1. Intervention: 113.2 to 94.2   Control: 125.2 to 116.8  *P* <0.05 |
| Clifford (2012), New South Wales | Qualitative  (Acceptability) | Sample size: number of clients not reported  Mean age: NR  Gender (male): NR  Substance use: Alcohol | Screening and Brief Intervention (SBI):  A cost-effective treatment for reducing alcohol consumption among drinkers in primary care  Alcohol screening independent of health assessments was generally selective; prompted by alcohol-related presentations, such as injuries and stress-related conditions, as well as disease and illness likely to be caused or worsened by heavy drinking. Brief intervention often includes strategies such as psychoeducation and motivational interviewing.  Duration: NR  Frequency: NR  Delivered by: health care workers (GPs, nurses, Aboriginal health workers)  Mode: individual, primary care clinic | Healthcare practitioners’ experiences and perspectives of Factors influencing the delivery of SBI | - GPs expressed concern that asking a patient about their alcohol use would identify complex problems they did not have the time or expertise to treat. - concerns that it could offend patients and damage rapport - how well alcohol SBI fitted within their role influenced their willingness to deliver it - practitioners’ utilisation of clinical systems and processes was low - alcohol SBI was not routinely or uniformly documented - Indigenous-specific alcohol SBI guidelines and resources, although available in all ACCHSs, were referred to infrequently - a lack of appropriate alcohol referral options was identified as a prominent barrier |
| Conigrave (2012), New South Wales | Mixed  (Acceptability) | Sample size: 58  Mean age: Not reported, 78% were aged 35 or over  Gender (male): 45%  Substance use: Alcohol | Alcohol Awareness intervention:  Screening, group education and brief intervention were delivered to various existing community-based groups. Facilitator was matched to the group’s gender (i.e. male facilitator would lead if predominantly all-male group). Sessions fitted within the normal activity of the host group (i.e. cooking, artwork). Participants engaged in screening (i.e. completion of the AUDIT) and provided with education (i.e. knowledge of drinking limits, treatment services etc) and brief intervention (i.e. identifying benefits of reducing alcohol intake, motivational interviewing).  Duration: 11-month period  Frequency: 9 sessions  Delivered by: male and female Aboriginal facilitator  Mode: Group, outreach | 1. AUDIT scores  2. Feasibility and acceptability | 1. 45% scored indicating potential problem drinking (i.e. drinking at risky levels, experiencing harm or dependence) 2. Groups of males were typically harder to engage  - considerable surprise (and sometimes amusement) was expressed at how low current recommended limits are – no participants seemed to be aware of drinking guidelines - No participants agreed to one-on-one brief intervention |
| D’Abbs (2013), Northern Territory | Quasi-experimental  (Impact) | Sample size: 129  Mean age: 38  Gender (male): 51%  Substance use: Alcohol | The Grog Mob intervention:  This multidisciplinary program comprised of three streams of care in its service model to address alcohol use: (i) pharmacotherapy; (ii) psychological therapy, including cognitive behavioural therapy, goal-setting, motivational interviewing and problem solving skills with individuals and families and (iii) social and cultural support which included working with supported employment and accommodation services, assisting Aboriginal people to explore their cultural roots and issues of Aboriginal identity while in treatment, and provide support to clients to resolve interpersonal and other conflicts.  Duration: eight weeks  Frequency: weekly  Delivered by: GP (pharmacotherapy), psychologist or social worker (therapy) and Aboriginal Liaison Officer who was a local language speaker (social and cultural support)  Mode: Individual, outreach support, capacity to involve families | 1. Alcohol use: self-report | Intervention: 79% reported they had stopped drinking or reduced their intake  Control: 70% reported they had stopped drinking or reduced their intake  Not statistically significant, and low sample size to provide confidence in the findings. |
| Daly (2021), New South Wales | Cross-sectional  (Impact and Acceptability) | Sample size: 100  Mean age: 26  Gender (male): 0%  Substance use: Tobacco | Smoking cessation support:  Smoking cessation support for pregnant Aboriginal women attending AMIHS for antenatal care. The AMIHS service delivery model includes home visiting outreach support and community-based clinics. During their antenatal visits, women had their smoking status assessed, were offered a Quitline referral, nicotine-replacement therapy, and follow-up support.  Duration: 12 month period  Frequency: each antenatal visit  Delivered by: midwife and Aboriginal health worker  Mode: Individual, outreach and antenatal clinic | 1. Acceptance of support  2. Factors associated with acceptance of support  3. Quitting behaviours | 1. a). 98% had their smoking status assessed   b) 86% offered Quitline referral; 35% accepted  c). 69% offered nicotine replacement therapy; 68% accepted; 44% used it for more than 8 weeks  d). 59% offered follow-up support; 56% accepted   - attending five or more antenatal visits - lack of interest in quitting smoking - beliefs that the support would not be helpful - wanting to quit without assistance  1. 63% quit for one day or more   66% quit two or more times  Mean time without a cigarette was 3 months  35% abstained for a month or more |
| Eades (2013),  Queensland and Western Australia | Randomised controlled trial  (Impact) | Sample size: 263  Intervention = 148  Control = 115  Mean age: Not reported  Gender (male): 0%  Substance use: Tobacco | Intensive quit-smoking intervention for pregnant Aboriginal women:  A general practitioner and other health care workers delivered tailored advice and support to quit smoking to women at their first AMIHS antenatal visit, using evidence-based communication skills and engaging the woman’s partner and other adults in supporting the quit attempts. The AMIHS service delivery model includes home visiting outreach support and community-based clinics. Follow-up was conducted by female Aboriginal or Torres Strait Islander health workers and midwives at subsequent visits. Nicotine replacement therapy was offered after two failed attempts to quit.  Duration: 36 weeks, first to last antenatal visit  Frequency: weekly  Delivered by: GP and Aboriginal health workers or midwives  Mode: Individual, outreach and clinics  Control group: received advice to quit from GP | % of smoking rates (validated by a urine cotinine measurement) | Intervention: 89% reported smoking at 36 weeks gestation  Control: 95% reported smoking at 36 weeks gestation  No significant difference between groups. The intensive quit-smoking intervention was no more effective than usual care in assisting pregnant Aboriginal and Torres Strait Islander women to quit smoking during pregnancy. |
| Fitts (2016), Queensland | Qualitative  (Acceptability) | Sample size: 17  Mean age: 62% aged between 26-39 years  Gender (male): 53%  Substance use: Alcohol | Hero to Healing drink driving program  Group program underpinned by the Community Reinforcement Approach. Four sessions focused on the impact of drink driving, issues relating to kinship pressures, risk taking, Indigenous values, general alcohol problems, and alcohol and cannabis education. Content is discussed through DVDs of community members discussing their experiences, illustrations, visual media, storytelling, yarning and interactive discussions without the need for participants to engage with written information. The program focuses on the implications of drink driving on the community and changing external factors to make not drinking more appealing. Strategies of learning to refuse a drink and drink driving (behavioural skills training), job skills, non-alcohol related social activities, support from a family member (buddy system) and relapse-prevention planning were discussed.  Duration: 4 weeks  Frequency: weekly 2-hour sessions  Delivered by: Community Elders and local drug and alcohol workers  Mode: group program and outreach | Participants perceptions of the feasibility and acceptability of the program | - participants identified/connected with the content - learning from Elders - safe environment: ‘on country’ and away from the community was deemed appropriate due to sensitivity of content - appealed to participants because of its flexible nature and encouragement of rearranging lifestyle factors, without specific focus on alcohol use   Recommendations:   - more in-depth discussion about other social and psychological aspects of drinking - presence of other external agencies at the program to provide participants with new training or employment opportunities upon program completion |
| Ivers (2003), Northern Territory | Quasi-experimental  (Impact) | Sample size: 111  Intervention = 40  Control = 71  Mean age: 50% over 38 years of age  Gender (male): 54%  Substance use: Tobacco | Brief intervention for smoking cessation and nicotine patches:  The brief intervention involved being given advice on quitting (including being given advice on the health effects of tobacco use, support in setting a quit date and counselling on cessation according to readiness to quit), being shown a flip-chart about tobacco and being offered a pamphlet. Participants were also instructed in the use of nicotine patches according to the manufacturer’s instructions.  Duration: 1 session of brief intervention, 10 weeks of nicotine patches  Frequency: 1 session of brief intervention, nicotine patches worn 24 hours a day  Delivered by: health worker, not specified  Mode: Individual  Control group: brief intervention only | Post-intervention outcomes assessed at 6 months.  1. Smoking cessation  2. Reduction in tobacco consumption  3. Readiness to quit | 1. Intervention: 15% reported quitting   Control: 1% reported quitting   1. Intervention: 76% reported reduced tobacco consumption   Control: 51% reported reduced tobacco consumption   1. No significant changes in readiness to quite between groups |
| Lee (2013), New South Wales | Qualitative  (Acceptability) | Sample size: 24 clients; 21 staff  Mean age: 29.5 years  Gender (male): 0%  Substance use: 87% opioids, 17% cannabis, 17% tobacco, 4% alcohol, 4% benzodiazepines, 4% stimulants | Aboriginal women’s support group:  Delivered as part of a multidisciplinary outpatient alcohol and other drug treatment service. The group is conducted in a large dedicated Aboriginal space alongside counselling rooms. The group format changes depending on the number of clients and demand but includes informal conversation, art and craft, and recreational and educational activities (e.g. on treatment options, parenting, first aid or financial management). Children are welcome, and lunch is provided. It is delivered by two female facilitators. Aboriginal health promotion, health education, early childhood and mental health workers employed by the same health district periodically attend as support staff. A non-Aboriginal volunteer assists with child minding.  Duration: Not reported  Frequency: 1x3-hour session, weekly  Delivered by: two female staff members: a senior Aboriginal woman with experience in AOD work and a non-Aboriginal counsellor  Mode: Group | Client and staff perspectives of the support group. | Perceived usefulness/strengths:   - an alternative to substance use - sense of stability and structure - relaxation and socialisation - share experiences and receive support in a family atmosphere, safe environment - received practical support to address key issues (e.g. financial, housing, child protection etc) - improved problem solving skills, confidence, self-efficacy - positive role modelling - pathway to early intervention and treatment   Suggested improvements:   - more opportunities to share stories, in line with Aboriginal traditions of storytelling - more staff from Aboriginal community (e.g. elders, artists, peer workers – mothers who have successfully completed AOD treatment) |
| Marley (2014), Western Australia | Randomised controlled trial  (Impact) | Sample size: 168  Intervention = 58  Control = 110  Mean age:  Intervention = 41.9 (11.9)  Control = 38.3 (12)  Gender (male): 47%  Substance use: Tobacco | Intensive smoking cessation intervention:  Aboriginal researchers (health worker background) delivered tailored smoking cessation counselling during face-to-face visits. Support included: motivational interviewing; diversions and strategies to deal with smoking triggers; action plans for preventing and dealing with short term relapses; discussion regarding the positives of smoking cessation; referral for and titration of pharmacotherapy; identification of factors driving smoking and case management to address these by linking participants with additional non-health support agencies (e.g. public housing, welfare, domestic violence and alcohol services); and strategies for smoking cessation- associated weight gain. They were also encouraged to attend a monthly smoking cessation peer support group.  Duration: 12 months  Frequency: weekly for the first month, monthly to six months, and bimonthly to 12 months  Delivered by: Aboriginal researchers  Mode: Individual, outreach and clinic visits  Control group: usual care smoking cessation from local primary care service | Outcome measured 12 months post enrolment.  % of smoking cessation: self-report and validated by urine cotinine | Intervention: 11%  Control: 5%  No significant difference, not a big enough size to be confident in the findings. |
| Nagel (2009), Northern Territory | Randomised controlled trial  (Impact) | Sample size: 49  Intervention = 24  Control = 25  Mean age: 33  Gender (male): 57%  Substance use: Mental illness with comorbid substance use, 65% cannabis, 63% alcohol 47% both cannabis and alcohol | Brief intervention and motivational care planning (MCP):  Treatment consisted of two one-hour treatment sessions, which integrated problem-solving, motivational therapy and self-management principles. MCP involved four steps: discussion about family support, exploration of strengths, identification of stressors, and goal setting. The second session, two to six weeks later, reviewed progress and developed new strategies as appropriate. The intervention incorporated family through engagement of carers in the treatment sessions, incorporation of carers on a ‘family map’, and by involving family in the goal-setting phase of the care-planning. Two brief psycho-educational videos were shown in each session with distribution of matching handouts.  Duration: two to six weeks  Frequency: 2x50 minute sessions  Delivered by: Aboriginal research officer, health worker, and local Aboriginal mental health worker  Mode: Individual with family involvement  Control group: waitlist control group | 1. Cannabis use: Severity of Dependence Scale  2. Alcohol use: Severity of Dependence Scale  3. Mental health functioning: Health of the Nations Outcome Scale  4. General functioning/life skills: Life Skills Profile  5. Psychological distress: Kessler-10 | No significant differences on any outcome scale.  May be due to small sample size. |
| Wearne (2005),  Northern  Territory | Qualitative  (Acceptability) | Sample size: NR  Mean age: NR  Gender (male): NR  Substance use: Alcohol | Community outreach program:  Targeted to heavy drinkers living in town camps.  Delivered by Miwatj Health. Sought to provide appropriate health care and treatment for drinkers, while responding to their various needs as they arose (e.g. informal individual and group counselling, a lift into town, or practical support that could take an entire working day). Aimed to facilitate screening and treatment by reaching people in the community and bringing them into the clinic for follow-up. They also regularly brought vitamin-B tablets and water to the camp sites.  Duration: NR  Frequency: five days a week, four hours a day  Delivered by: Two Indigenous program coordinators  Mode: Outreach | Client and staff experiences and perspectives of the program; feasibility | *Acceptable:*   - qualitative reports of helping some individuals curtail their dependent drinking - developed relationships with staff - reconnection to family, kin and community networks - restoring links with families, cultural and ceremonial obligations, sport and recreation - hunting trips were valued to reconnect to culture and social networks, and a diversion from drinking - helped form peer groups that devalue drug and alcohol abuse   *Areas for improvement:*   - difficult to encourage some participants to attend the clinic - isolated from the broader organisation - staff lacked professional support - more cultural engagement - increase participation and guidance of local Aborigines as leaders in the program |
| Williams (2006), South Australia | Mixed methods  (Impact) | Sample size: 88  Mean age: NR  Gender (male): 52%  Substance use: Opioids | The Way Out Program:  A multi-faceted program that offers drug treatment (opioid substitution therapy) within a family-friendly holistic primary care service. It locates the drug problem within a holistic view of the individual’s health. The strong community linkages and family support through case management and community-based activity programmes complement the pharmacological support, counselling and brokerage assistance. ‘Healing days’ were held for families affected by drug abuse to expose the community to an eclectic mix of therapies for healing and relaxation including massage, meditation, crystals, traditional healers and more. The role of detoxification and methadone maintenance is explained as components of treatment, rather than as cures. Provided home detox ‘kits’ (an easy-to-use package consisting of basic information and 3 days’ supply of symptomatic relief medication) and written resources.  Duration: NR  Frequency: NR  Delivered by: Indigenous drug and alcohol worker, two GPs  Mode: Outreach, whole-family support | Client and staff experiences and perspectives of the program  Rate of substitution | 10% successful completion  40% current stabilisation  34% Default/relapse before or after stabilisation  16% referred on  Qual:   - engaging and educating community leaders - trust and relationships formed between workers and clients - emphasised that detoxification was not a cure for heroin addiction - provides services or facilities available for rehabilitation after physical withdrawal - large local Aboriginal community presence - Aboriginal-specific resources - caters to clients’ needs - established strong links with correctional services |

AMIHS, Aboriginal Maternal and Infant Health Services; AUDIT, Alcohol Use Disorders Identification Test; GP, general practitioner; NR, not recorded; SBI, Screening and Brief Intervention.
